# Unexplained Giant Genital Enlargement: Is It Due to Inverse Psoriasis?

**DOI:** 10.3390/reports7040092

**Published:** 2024-11-07

**Authors:** Francesco Natale, Giovanni Cimmino

**Affiliations:** 1 Vanvitelli Cardiology and Intesive Care Unit, Monaldi Hospital, 80131 Naples, Italy; 2 Cardiology Unit, Azienda Ospedaliera Universitaria Luigi Vanvitelli, 80138 Napoli, Italy; 3 Department of Translational Medical Sciences, Section of Cardiology, University of Campania Luigi Vanvitelli, 80131 Naples, Italy

**Keywords:** genital swelling, inverse psoriasis, lymphoscintigraphy, elephantiasis

## Abstract

A healthy 54-year-old man previously presented to vascular surgeons with a 4-year history of swelling of the penis and scrotum was scheduled for ultrasound evaluation in the angiology office in our department. At presentation, there was a giant enlargement of the penis and scrotum, without swelling of the legs. Ultrasound evaluation was negative for vascular abnormalities. A diagnosis of chronic lymphatic disease was suspected; thus, a lymphoscintigraphy was performed. This test was normal showing, a good visualization of major lymphatics. The patients had a history of psoriasis with a documented previous event of flexural psoriasis involving his genitals with secondary infection 4 years before. Since that infection, his genitals progressively increased in size, and despite medical treatment and different surgical evaluations, the patient’s symptoms have not resolved, with marked disability associated with walking and sexual activity.

Penoscrotal elephantiasis (PSE) is a rare condition characterized by an increased volume of the external genitalia [1]. This enlargement is sometimes considerable, with a great sexological and psychological impact [2]. It is usually related to an intrinsic or extrinsic lymphatic obstruction that may be primary or secondary to an underlying disease [3]. Filarsiosis is one of the most commonly associated infectious diseases [4]. Other causes may be cancer, inflammation and granulomatous processes, chronic infection, radiotherapy, hydroelectrolytic disbalances, and idiopathy [5]. The diagnosis is mainly made by physical examination. Pharmacological treatment of filariasis, when it is diagnosed, is well established; however, a surgical approach to genital elephantiasis is sometimes necessary, despite the varied and confusing strategies [6]. Ideally, surgical treatment should consist of mass excision and reconstruction using healthy skin. Genital skin can also be affected by inverse or flexural psoriasis with considerable discomfort, embarrassment and impairments in quality of life and sexual activity [7]. These skin lesions might be a point of entry for infectious agents. Here, we report a case of penoscrotal enlargement, recognizing the possible cause of the inverse psoriasis (Figure 1). 

Flexural psoriasis, also called inverse psoriasis, describes psoriasis localized to the skin folds and genitals [8]. Due to the moist nature of the skin folds, the appearance of the psoriasis was slightly different. It was shiny and smooth with a crack (fissure) in the depth of the skin crease. A deep-red color and well-defined borders characteristic of psoriasis were obvious (Figure 1A, white arrows).

This case itself is unique, since after 4 years of investigation, no other causes of penis enlargement have been found. The inverse psoriasis seems to be linked to the swelling caused by the associated skin infection. It is important to seek treatment right away if an infection is suspected. A careful evaluation of injured skin in psoriatic patients is of great importance to avoid serious complications.

## Figures and Tables

**Figure 1:**
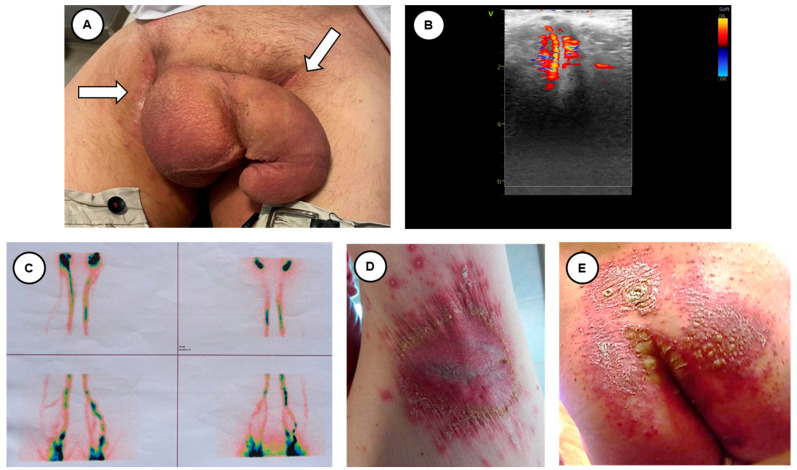
A healthy 54-year-old man previously presented to vascular surgeons with a 4-year history of swelling of the penis and scrotum was scheduled for ultrasound evaluation in the angiology office. At presentation, there was a giant enlargement of the penis and scrotum, without swelling of the legs (**A**). The penile ultrasound was negative for vascular abnormalities and/or thrombotic complications (**B**). A diagnosis of chronic lymphatic disease was suspected; thus, a lymphoscintigraphy was performed (**C**). This test was normal, showing a good visualization of major lymphatics. The patient had a history of psoriasis, with a documented previous event of flexural psoriasis, as shown in (**D**) (axillary fossa) and (**E**) (left and right gluteus and intergluteal cleft), also involving his genitals with secondary infection 4 years before. Since that infection, his genitals progressively increased in size, and despite medical treatment and different surgical evaluation, the patient’s symptoms did not resolve, with marked disability related to walking and sexual activity. The diagnostic work-up for filariasis was performed as soon as the genital enlargement started, with negative results (direct detection in the blood, antigen detection and molecular diagnosis by PCR). The patient reported that, before experiencing penis enlargement, an acute phase of psoriasis occurred with signs of infection, as diagnosed by the family practitioner, and because of that, empirical antibiotic treatment was started. Taking into account the negative results of the test for filariasis, and the absence of vascular obstruction (as shown by the Doppler evaluation) as well as lymphatic obstruction (as shown by the lymphoscintigraphy), the role of inverse psoriasis in generating this process was postulated.

## Data Availability

The data underlying this study are included in the article, further inquiries can be directed to the corresponding author.
